# Impact of sarcopenic obesity on functional outcomes

**DOI:** 10.18632/aging.204549

**Published:** 2023-02-23

**Authors:** Akio Shimizu, Tatsuro Inoue, Keisuke Maeda

**Affiliations:** 1 Department of Health Science, Faculty of Health and Human Development, The University of Nagano, Nagano, Japan; 2 Department of Geriatric Medicine, Hospital, National Center for Geriatrics and Gerontology, Aichi, Japan; 3 Department of Physical Therapy, Niigata University of Health and Welfare, Niigata, Japan

**Keywords:** obesity, ESPEN and EASO, sarcopenic obesity, functional outcomes, visceral fat

Sarcopenic obesity (SO) has recently become a new concept that has received attention in many disciplines, including geriatrics. SO is associated with functional decline and increased mortality [1], hence prevention and therapeutic intervention are important. There are no consistent diagnostic criteria for SO as there are several diagnostic criteria and methods for SO worldwide. Adapting different criteria for the diagnosis of SO may adversely affect its management. To address these issues, in 2022, the European Society for Clinical Nutrition and Metabolism (ESPEN) and the European Association for the Study of Obesity (EASO) proposed unified diagnostic criteria [1].

The ESPEN and EASO SO criteria consist of three steps: screening, diagnosis, and severity grading. First, SO screening evaluates the presence of a high waist circumference or body mass index (BMI), symptoms, clinical suspicion of sarcopenia, or a questionnaire (e.g., SARC-F). Second, the diagnostic step evaluates several items related to SO. Muscle functional parameters were assessed using handgrip strength or the chair stand test. Body composition was evaluated using dual X-ray absorptiometry (DXA) or bioelectrical impedance analysis (BIA). Body composition assessment included increased body fat mass (body fat %) and reduced muscle mass (appendicular lean mass/body weight on DXA or skeletal muscle mass/body weight on BIA). SO is diagnosed when low muscle function, increased body fat mass, and reduced muscle mass are all present. Finally, cases without functional disabilities or endocrine excess were classified as stage 1 SO, and those with it were classified as stage 2 SO. The ESPEN and EASO criteria provide cutoff values for age, sex, and ethnicity based on previous studies [1].

SO is thought to influence functional decline and disability incidence [1]. However, there are no consistent findings on whether SO diagnosed using ESPEN and EASO criteria negatively affects functional outcomes [2, 3]. Two studies diagnosed SO using the diagnostic parameters of high BMI, low handgrip strength, increased body fat mass, and reduced skeletal muscle mass/body weight by BIA. Yoshimura et al. [3] retrospectively investigated the association between SO diagnosed according to the ESPEN and EASO criteria and functional outcomes in 760 Japanese patients with stroke requiring rehabilitation (median age 73 y, 46.3% female). The prevalence of SO was 4.5% (4.1% in men and 5.4% in women) and had a negative impact on functional outcomes. Similarly, we retrospectively investigated the association between SO diagnosed with the ESPEN and EASO criteria and functional outcomes in 1080 older Japanese patients requiring rehabilitation (mean age 79.5 y, 56.5% females) [2]. The prevalence of SO was 4.5–5.3% (4.0–5.7% in men and 4.4–4.9% in women) after adapting several cutoff values proposed by ESPEN and EASO. However, our study showed no association between SO and functional outcomes. These studies may not have been adequately adjusted for confounding factors because they were retrospective. It is also important to note that these studies only included Asians. Therefore, future prospective studies should be conducted in various ethnic groups.

Several considerations exist to clarify whether SO diagnosed using ESPEN and EASO criteria adversely affects functional outcomes. First, it is necessary to consider whether waist circumference or BMI is the preferred definition of obesity. Previous studies have not yielded consistent results regarding whether obesity, as defined by BMI, worsens functional outcomes in patients [4, 5]. Obesity, as defined by BMI, has a negative impact on functional outcomes in patients requiring surgical treatment for ankle fractures [4]. Interestingly, a U-shaped association between BMI and functional outcomes may exist in patients with ischemic stroke [5]. This finding suggests that there may be an “obesity paradox” in functional outcomes. However, no studies have compared SO diagnosed using ESPEN and EASO based on waist circumference. Waist circumference is a good marker for visceral fat and skeletal muscle and visceral fat have similar inflammatory pathways [6]. In addition, visceral fat is thought to cause sarcopenia by inducing inflammation and adversely affecting the skeletal muscle. Therefore, high visceral fat may negatively impact functional outcomes more than a high BMI. Further studies are required to determine the influence of waist circumference on SO. Second, an optimal muscle mass adjustment method must be considered. The ESPEN and EASO criteria suggest adjusting muscle mass for body weight [1]. However, adjusting muscle mass according to body weight is considered inadequate to account for the body size of obese individuals [7]. Bahat et al. [7] proposed adjusting the muscle mass using BMI to solve this problem. A previous study reported that adjusting for muscle mass by BMI predicted functional outcomes better than adjusting for muscle mass by height or weight [7]. In addition, it has been shown that adjusting for muscle mass by BMI is a stronger predictor of the risk of falls than adjusting for muscle mass by height in older Japanese individuals [8]. Therefore, it may be desirable to use a method that adjusts for muscle mass by BMI for SO diagnosis using ESPEN and EASO. Finally, distinct cut-off values based on age, sex, and ethnicity should be determined for the ESPEN and EASO SO components. The ESPEN and EASO criteria propose cut-off values based on previous studies [1]. However, each cut-off value is not optimized for the ESPEN and EASO criteria. Thus, they may not be optimal cut-off values for diagnosing SO.

In conclusion, resolving these considerations is important for adapting SO diagnosis to different settings using ESPEN and EASO criteria. Further high-quality research on the impact of SO diagnosed using ESPEN and EASO on functional outcomes is warranted.
